# Supplementary material for: OrtSuite: from genomes to prediction of microbial interactions within targeted ecosystem processes
Source: Life Sci Alliance. 2021 Sep 27;4(12):e202101167. doi: 10.26508/lsa.202101167 (PMC8500227; doi:10.26508/lsa.202101167)
Supplement: Supplementary file 1 [file LSA-2021-01167_TableS1.docx]

Table S1 - Example of user-defined constraints. Definition of pathway name, complete set of reactions present in each pathway, sets of reactions required to be performed by single species (each subset is described between parenthesis) and transport reactions. Transporter column describes the transport reaction (e.g. R00750) that is coupled to the reaction in the pathway (e.g. R02601). Thus, species that perform the latter must also contain the genes associated to the transport reaction.

| Pathway | Reactions | Single_org | Transporter |
| --- | --- | --- | --- |
| Anaerobic benzoate-acetyl-CoA | R02601,R00813,R02604,R00816 | (R02601,R02604),(R00813,R00816) | (R00750:R02601) |
| Aerobic benzoate-acetyl-CoA | R02601,R00750,R00813 |  |  |
