# Supplementary material for: OrtSuite: from genomes to prediction of microbial interactions within targeted ecosystem processes
Source: Life Sci Alliance. 2021 Sep 27;4(12):e202101167. doi: 10.26508/lsa.202101167 (PMC8500227; doi:10.26508/lsa.202101167)
Supplement: Supplementary file 2 [file LSA-2021-01167_TableS2.docx]

Table S2 - Sequence alignments of original (query acc.ver) and mutated sequences (subject acc.ver) using BLAST *(Altschul* et al*, 1990)*. (eba: *Aromatoleum aromaticum* EbN1; azi: *Azoarcus* sp. CIB; bvi: *Burkholderia vietnamiensis* G4)

| query acc.ver | subject acc.ver | % identity | e-value | bit score | % positives |
| --- | --- | --- | --- | --- | --- |
| CAI09134.1 | eba1:CAI09134.1 | 98.592 | 0 | 721 | 99.15 |
| CAI09134.1 | eba3:CAI09134.1 | 94.366 | 0 | 687 | 96.06 |
| CAI09134.1 | eba5:CAI09134.1 | 92.58 | 0 | 532 | 95.76 |
| CAI09134.1 | eba15:CAI09134.1 | 79.532 | 2.98e^-102^ | 288 | 88.3 |
| CAI09134.1 | eba10:CAI09134.1 | 84.252 | 4.27e^-74^ | 215 | 88.98 |
| CAI09134.1 | eba25:CAI09134.1 | 64 | 5.36e^-13^ | 51.6 | 80 |
| AKU14370.1 | azi1:AKU14370.1 | 98.828 | 0 | 507 | 99.22 |
| AKU14370.1 | azi3:AKU14370.1 | 94.531 | 5.98e^-180^ | 484 | 96.09 |
| AKU14370.1 | azi5:AKU14370.1 | 91.797 | 1.25e^-174^ | 470 | 94.92 |
| AKU14370.1 | azi15:AKU14370.1 | 74.775 | 1.37e^-116^ | 322 | 82.43 |
| AKU14370.1 | azi10:AKU14370.1 | 87.705 | 2.26e^-76^ | 216 | 94.26 |
| AKU14370.1 | azi25:AKU14370.1 | 64.22 | 8.78e^-45^ | 135 | 75.23 |
| ABO58758.1 | bvi1:ABO58758.1 | 98.837 | 0 | 508 | 98.84 |
| ABO58758.1 | bvi3:ABO58758.1 | 96.296 | 4.12e^-116^ | 318 | 98.15 |
| ABO58758.1 | bvi5:ABO58758.1 | 90.184 | 4.99e^-109^ | 301 | 91.41 |
| ABO58758.1 | bvi10:ABO58758.1 | 81.595 | 6.90e^-97^ | 270 | 86.5 |
| ABO58758.1 | bvi25:ABO58758.1 | 61.321 | 3.56e^-41^ | 126 | 67.92 |
| ABO58758.1 | bvi15:ABO58758.1 | 81.818 | 2.54e^-40^ | 123 | 81.82 |
