# Supplementary material for: OrtSuite: from genomes to prediction of microbial interactions within targeted ecosystem processes
Source: Life Sci Alliance. 2021 Sep 27;4(12):e202101167. doi: 10.26508/lsa.202101167 (PMC8500227; doi:10.26508/lsa.202101167)
Supplement: Supplementary file 4 [file LSA-2021-01167_TableS4.docx]

Table S4 – Overview of OrtSuite results using the Test_genome set using an E-value of 0.001 during the relaxed search and four different E-values during the restrictive search (1e^-4^, 1e^-6^, 1e^-9^ and 1e^-16^): number of orthogroups, number of KEGG orthologs (KO) in ORAdb and the number of ortholog clusters that transition from each annotation phase, number of consistent (ConOG) and divergent (DivOG) orthogroups.

| E-value | 1e^-4^ | 1e^-6^ | 1e^-9^ | 1e^-16^ |
| --- | --- | --- | --- | --- |
| Total Orthogroups | 52648 | 52648 | 52648 | 52648 |
| KOs in the database | 55 | 55 | 55 | 55 |
| (Relaxed Search) Selected orthogroups | 460 | 460 | 452 | 443 |
| (Relaxed Search) % Selected orthogroups | 0.9 | 0.9 | 0.9 | 0.8 |
| (Relaxed Search) Associated KOs | 52 | 52 | 53 | 53 |
| (Relaxed Search) % Associated KOs | 94.5 | 94.5 | 96.4 | 96.4 |
| (Restrictive Search) Orthogroups with annotated sequences | 371 | 369 | 351 | 285 |
| (Restrictive Search) % of Orthogroups with annotated sequences | 0.7 | 0.7 | 0.7 | 0.5 |
| (Restrictive Search) KOs with assigned sequences | 52 | 52 | 52 | 52 |
| (Restrictive Search) % KOs with annotated sequences | 94.5 | 94.5 | 94.5 | 94.5 |
| ConOG | 149 | 150 | 149 | 131 |
| DivOG | 222 | 219 | 202 | 154 |
| DivOG with more than one KO | 104 | 104 | 97 | 87 |
| **Relaxed Search to Restrictive Search** |  |  |  |  |
| Lost orthogroups | 89 | 91 | 101 | 158 |
| % Lost orthogroups | 19.3 | 19.8 | 22.3 | 35.7 |
| Lost KOs | 0 | 0 | 1 | 1 |
| % Lost KOs | 0 | 0 | 1.9 | 1.9 |
