# Supplementary material for: OrtSuite: from genomes to prediction of microbial interactions within targeted ecosystem processes
Source: Life Sci Alliance. 2021 Sep 27;4(12):e202101167. doi: 10.26508/lsa.202101167 (PMC8500227; doi:10.26508/lsa.202101167)
Supplement: Supplementary file 5 [file LSA-2021-01167_TableS5.docx]

Table S5 – Performance of OrtSuite in functional annotation of the Test_genome_set using four different e-value cutoffs (1e^-04^,1e^-06^,1e^-09^,1e^-16^). Values represent the percentage of KEGG annotations in the Test genome set confirmed by OrtSuite.

| E-value | 1e^-04^ | 1e^-06^ | 1e^-09^ | 1e^-16^ |
| --- | --- | --- | --- | --- |
| *Acinetobacter defluvii* WCHA30 | 100 | 100 | 100 | 100 |
| *Arabidopsis thaliana* | 100 | 100 | 100 | 100 |
| *Azoarcus sp.* KH32C | 100 | 100 | 100 | 100 |
| *Azoarcus sp*. DN11 | 100 | 100 | 100 | 100 |
| *Azoarcus sp.* CIB | 100 | 100 | 100 | 100 |
| *Burkholderia cepacia* DDS 7H-2 | 88.88 | 88.88 | 88.88 | 88.88 |
| *Burkholderia vietnamiensis* G4 | 90.90 | 90.90 | 86.36 | 86.36 |
| *Cycloclasticus sp.* P1 | 86.66 | 86.66 | 86.66 | 86.66 |
| *Cycloclasticus zancles* 78-ME | 87.5 | 87.5 | 87.5 | 87.5 |
| *Desulfosporosinus orientis* DSM 765 | 92.30 | 92.30 | 92.30 | 92.30 |
| *Aromatoleum aromaticum* EbN1 | 100 | 100 | 100 | 100 |
| *Latimeria chalumnae* (coelacanth) | 100 | 100 | 100 | 100 |
| *Magnetospirillum sp.* XM-1 | 100 | 100 | 100 | 100 |
| *Paraburkholderia aromaticivorans* BN5 | 100 | 100 | 100 | 100 |
| *Rhodococcus ruber* P14 | 100 | 100 | 100 | 100 |
| *Sulfuritalea hydrogenivorans* sk43H | 100 | 100 | 100 | 100 |
| *Staphylococcus sciuri* FDAARGOS 285 | 85.71 | 85.71 | 71.42 | 85.71 |
| *Thauera sp.* MZ1T | 100 | 100 | 100 | 100 |
| **Average** | **96.22** | **96.22** | **95.18** | **95.96** |
