# Supplementary material for: OrtSuite: from genomes to prediction of microbial interactions within targeted ecosystem processes
Source: Life Sci Alliance. 2021 Sep 27;4(12):e202101167. doi: 10.26508/lsa.202101167 (PMC8500227; doi:10.26508/lsa.202101167)
Supplement: Supplementary file 14 [file LSA-2021-01167_TableS14.docx]

Table S14 – Benzoate degradation pathways used for evaluation of OrtSuite. Description of each alternative pathway, abbreviation code and reaction identifiers.

| Pathway | Abbreviation code | Reactions |
| --- | --- | --- |
| Anaerobic benzoate-acetyl-CoA_1 | P1 | R01422,R02451,R05597,R05581,R05594,R05305,R05586,R05579,R03028,R03026,R01976,R00238 |
|  |  |  |
| Anaerobic benzoate-acetyl-CoA_2 | P2 | R01422,R02451,R05597,R05581,R05594,R05305,R05586,R02488,R03026,R01976,R00238 |
|  |  |  |
| Aerobic benzoate-acetyl-CoA | P3 | R05621,R00813,R00816,R02604,R00750,R00228,R02601 |
