# Supplementary material for: OrtSuite: from genomes to prediction of microbial interactions within targeted ecosystem processes
Source: Life Sci Alliance. 2021 Sep 27;4(12):e202101167. doi: 10.26508/lsa.202101167 (PMC8500227; doi:10.26508/lsa.202101167)
Supplement: Supplementary file 15 [file LSA-2021-01167_TableS15.docx]

| Name and strain | Abbreviation code | KEGG | E-value | | | |
| --- | --- | --- | --- | --- | --- | --- |
|  |  |  | 1e^-4^ | 1e^-6^ | 1e^-9^ | 1e^-16^ |
| *Acinetobacter defluvii* WCHA30 | adv | P3 | P3 | P3 | P3 | P3 |
| *Arabidopsis thaliana* | ath | - | - | - | - | - |
| *Azoarcus sp.* KH32C | aza | P2 | P1,P2 | P1,P2 | P1,P2 | P1,P2 |
| *Azoarcus sp*. DN11 | azd | P2 | P3 | P3 | P3 | P3 |
| *Azoarcus sp.* CIB | azi | P2 | P1,P2,P3 | P1,P2,P3 | P1,P2,P3 | P1,P2 |
| *Burkholderia cepacia* DDS 7H-2 | bced | P3 | P3 | P3 | P3 | P3 |
| *Burkholderia vietnamiensis* G4 | bvi | P3 | P3 | P3 | P3 | P3 |
| *Cycloclasticus sp.* P1 | cyq | P3 | P3 | P3 | P3 | P3 |
| *Cycloclasticus zancles* 78-ME | cza | P3 | P3 | P3 | P3 | P3 |
| *Desulfosporosinus orientis* DSM 765 | dor | - | - | - | - | - |
| *Aromatoleum aromaticum* EbN1 | eba | P2 | P1,P2 | P1,P2 | P1,P2 | P1,P2 |
| *Latimeria chalumnae* (coelacanth) | lcm | - | - | - | - | - |
| *Magnetospirillum sp.* XM-1 | magx | P2 | P1,P2 | P1,P2 | P1,P2 | P1,P2 |
| *Paraburkholderia aromaticivorans* BN5 | parb | P3 | P3 | P3 | P3 | P3 |
| *Rhodococcus ruber* P14 | rrz | P3 | P3 | P3 | P3 | P3 |
| *Sulfuritalea hydrogenivorans* sk43H | shd | P2 | P1,P2 | P1,P2 | P1,P2 | P1,P2 |
| *Staphylococcus sciuri* FDAARGOS 285 | sscu | - | - | - | - | - |
| *Thauera sp.* MZ1T | tmz | P2, P3 | P3 | P3 | P3 | P3 |

Table S15 – OrtSuite identification of species from the *Test_genome_set* with the genomic potential to perform all reactions in each of the alternative benzoate to acetyl-CoA conversion pathways using different restrictive e-value cutoffs.
