# Supplementary material for: OrtSuite: from genomes to prediction of microbial interactions within targeted ecosystem processes
Source: Life Sci Alliance. 2021 Sep 27;4(12):e202101167. doi: 10.26508/lsa.202101167 (PMC8500227; doi:10.26508/lsa.202101167)
Supplement: Supplementary file 17 [file LSA-2021-01167_TableS17.docx]

Table S17 - Overview of the number of clusters, sequences and KOs during the annotation of the Fetzer_genome_set. (ConOG: consistent orthologs; DivOG: Divergent ortholog; KO: KEGG ortholog)

| Total Orthogroups | 31213 |
| --- | --- |
| **KOs in the database** | 55 |
| **(Relaxed Search) Selected orthogroups** | 417 |
| **(Relaxed Search) % Selected orthogroups** | 1.3 |
| **(Relaxed Search) Associated KOs** | 51 |
| **(Relaxed Search) % Associated KOs** | 92.7 |
| **(Restrictive Search) Orthogroups with annotated sequences** | 326 |
| **(Restrictive Search) % of Orthogroups with annotated sequences** | 1 |
| **(Restrictive Search) KOs with assigned sequences** | 47 |
| **(Restrictive Search) % KOs with annotated sequences** | 85.5 |
| **ConOG** | 92 |
| **DivOG** | 234 |
| **DivOG with more than one KO** | 139 |
| **Relaxed Search to Restrictive Search** |  |
| **Lost orthogroups** | 91 |
| **% Lost orthogroups** | 21.8 |
| **Lost KOs** | 4 |
| **% Lost KOs** | 7.8 |
