# Supplementary material for: OrtSuite: from genomes to prediction of microbial interactions within targeted ecosystem processes
Source: Life Sci Alliance. 2021 Sep 27;4(12):e202101167. doi: 10.26508/lsa.202101167 (PMC8500227; doi:10.26508/lsa.202101167)
Supplement: Supplementary file 21 [file LSA-2021-01167_TableS21.docx]

Table S21 – Number of species that contain all genes required to perform each reaction (KEGG RID) involved in the aerobic degradation of benzoate to acetyl-CoA (P3).

| **Reaction** | R05621 | R00813 | R00816 | R02604 | R00750 | R00228 | R02601 |
| --- | --- | --- | --- | --- | --- | --- | --- |
| **Number of Species** | 3 | 7 | 5 | 7 | 7 | 4 | 6 |
