# Supplementary material for: OrtSuite: from genomes to prediction of microbial interactions within targeted ecosystem processes
Source: Life Sci Alliance. 2021 Sep 27;4(12):e202101167. doi: 10.26508/lsa.202101167 (PMC8500227; doi:10.26508/lsa.202101167)
Supplement: Supplementary file 23 [file LSA-2021-01167_TableS23.docx]

Table S23 - Number of species that contain all genes required to perform each reaction involved in the anaerobic degradation of benzoate to acetyl-CoA (P1 and P2).

| **Reaction** | R00238 | R01422 | R02451 | R05597 | R05581 | R05594 | R05305 | R05586 | R05579 | R03028 | R03026 | R01976 | R02488 |
| --- | --- | --- | --- | --- | --- | --- | --- | --- | --- | --- | --- | --- | --- |
| **N° of Species** | 12 | 12 | 0 | 1 | 11 | 1 | 11 | 11 | 11 | 12 | 6 | 12 | 12 |
