# Supplementary material for: OrtSuite: from genomes to prediction of microbial interactions within targeted ecosystem processes
Source: Life Sci Alliance. 2021 Sep 27;4(12):e202101167. doi: 10.26508/lsa.202101167 (PMC8500227; doi:10.26508/lsa.202101167)
Supplement: Supplementary file 25 [file LSA-2021-01167_TableS25.docx]

Table S25 - Number of reactions, enzymes, KO groups and KO-associated sequences represented in each alternative benzoate to acetyl-CoA conversion pathway used (P1, P2 and P3). (P = pathway for Benzoate to Acetyl-CoA conversion)

|  | P1 | P2 | P3 |
| --- | --- | --- | --- |
| **Total Reactions** | 12 | 11 | 7 |
| **Total Enzymes** | 12 | 11 | 7 |
| **Total KO groups** | 32 | 31 | 14 |
